# Physical activity, sedentary behaviour, and sleep in the Thai population: A compositional data analysis including 135,824 participants from two national time-use surveys

**DOI:** 10.1371/journal.pone.0280957

**Published:** 2023-01-24

**Authors:** Nucharapon Liangruenrom, Dorothea Dumuid, Zeljko Pedisic

**Affiliations:** 1 Institute for Population and Social Research, Mahidol University, Phutthamonthon, Nakhon Pathom, Thailand; 2 Allied Health & Human Performance, Alliance for Research in Exercise, Nutrition and Activity, University of South Australia, Adelaide, Australia; 3 Centre for Adolescent Health, Murdoch Children’s Research Institute, Parkville, Victoria, Australia; 4 Institute for Health and Sport, Victoria University, Melbourne, Victoria, Australia; University of Study of Bari Aldo Moro, ITALY

## Abstract

**Purpose:**

To determine the amounts of time spent in physical activity (PA), sedentary behaviour (SB), and sleep in the Thai population, as well as their sociodemographic correlates and changes over time.

**Methods:**

We analysed cross-sectional data collected in a population-representative, stratified random sample of 135,824 Thais aged 10 years and over as part of the two most recent Thai National time-use surveys (2009 and 2015). Daily activities reported by the participants were coded using the International Classification of Activities for Time-Use Statistics (ICATUS) and categorised as PA, SB, or sleep.

**Results:**

In the latest survey, participants spent on average the largest amount of time sleeping (geometric mean [g] = 9.44 h/day; 95% confidence interval [CI]: 9.42, 9.47), followed by PA (g = 8.60 h/day; 95% CI: 8.55, 8.64) and SB (g = 5.96 h/day; 95% CI: 5.93, 6.00). The time spent in PA was higher on weekdays, while the amounts of SB and sleep were higher on weekends (p < 0.05). Males, older age groups, and unemployed people spent less time in PA and more time in SB, compared with other population groups (p < 0.05). We found a relatively large increase in SB (mean difference [d] = 39.64 min/day; 95% CI: 36.18, 42.98) and decrease in PA (d = 54.33 min/day; 95% CI: -58.88, -49.30) over time. These findings were consistent across most sociodemographic groups, with the most concerning shifts from active to sedentary lifestyle found among people with a higher education degree and on weekends.

**Conclusions:**

Our findings revealed a shift to a more sedentary lifestyle in the Thai population. Public health interventions should focus on improving time use among males, older age groups, and unemployed people, while preventing the rapid decrease in PA and increase in SB among those with a higher education degree and on weekends.

## Introduction

A growing body of evidence suggests that the time-use composition consisting of physical activity (PA), sedentary behaviour (SB), and sleep (often referred to as “24-hour movement behaviours”) influences a range of health outcomes at all ages [1–3]. These behaviours have significant impact on the population burden of disease. Systematic reviews consistently report beneficial effects of PA on health, including primary and secondary prevention of several chronic diseases, physical fitness, mental health and well-being [4–6]. There is strong evidence for unfavourable health consequences associated with SB, such as increased risk of premature mortality, depression, poor cognitive function, and chronic diseases such as cardiovascular disease, type 2 diabetes, and some cancers [7–11]. Insufficient, as well as too much, sleep has been linked with increased mortality and higher risk of NCDs such as cardiovascular disease, type 2 diabetes, hypertension, and obesity [12, 13].

Given that the amounts of time spent in these behaviours are by nature co-dependent and perfectly multi-collinear, any change in one behaviour must result in the net opposite change across the remaining behaviours [14–16]. The research paradigm has, therefore, shifted away from focusing on a single movement behaviour to the integration of all of these behaviours together. This shift has been facilitated by the application of an appropriate statistical methodology for dealing with time-use data—compositional data analysis (CoDA) [17].

CoDA is widely used in several areas of research, such as geology, economics, and epidemiology [14, 18]. It was developed to deal with compositional data which have specific inherent properties that cannot be represented appropriately in traditional statistical models. Compositional data are made up of mutually exclusive and exhaustive parts that always sum to a given total [14]. Because compositional data are ‘closed’ to a fixed sum, the constituent parts are co-dependent on each other and perfectly multi-collinear. In other words, it is impossible to change one part without also changing other part(s) to compensate. Thus, the data have a relative nature–a change to any one part must always be considered relative to the change in the other part(s). CoDA involves expressing the compositional data as a set of log-ratios that capture all the relative information about the composition. These log-ratios are then used in standard statistical models instead of the raw variables, allowing the interplay between variables to be accounted for, and avoiding statistical issues due to perfect multi-collinearity. Back-transformation of the log-ratios following statistical modelling allows the results to be interpreted in the original absolute scale. Movement behaviours are compositional data because they consist of times spent in PA, SB, and sleep, which always sum to 24 hours per day [14]. Because the times spent in PA, SB, and sleep are compositional data, CoDA is appropriate approach to analyse these behaviours in relation to each other and in relation to other variables (e.g. their correlates) [15].

A growing number of studies on PA, SB, and sleep are applying CoDA to account for the compositional properties of time-use data [19–36]. Such studies most commonly aim to determine correlates, determinants or health outcomes of the time-use composition. Other important topics according to the framework for *Viable Integrative Research in Time-Use Epidemiology* (VIRTUE) [16], including time trends in PA, SB, and sleep, have received much less attention. There also seems to be a disproportionately low number of time-use epidemiology studies from low- and middle-income countries.

Furthermore, methodological studies indicate that different measures may produce different estimates of PA, SB, and sleep [37] and that each measure has specific limitations [38, 39]. So far, most studies in time-use epidemiology have collected data on PA, SB, and sleep using accelerometers or a combination of sleep diaries and accelerometers. To confirm findings of such studies, more studies using other available measurement methods (e.g. time-use surveys, questionnaires) are needed. A recent scoping review that included 564 studies on PA and SB in Thailand concluded that more studies using large samples are needed to improve the generalizability of estimates [40].

Time-use surveys collecting comprehensive 24-hour information on daily activities have been conducted worldwide [41], and several methods are available for estimating the amounts of time spent in PA, SB, and sleep from such data [42–47]. Time-use surveys have adequate measurement properties for observational studies, with a high test-retest reliability comparable to that of accelerometer data [48]. Time-use surveys are, therefore, considered to be a valuable resource for studies on the full 24-hour spectrum of movement behaviours, specifically at a population level [49].

The National Statistical Office (NSO) in Thailand conducted national time-use surveys in 2001, 2004, 2009, and 2014/2015 [50–53]. This has provided an opportunity to examine changes in the time-use composition consisting of PA, SB, and sleep over time in the Thai population. Prior studies in Thailand have examined PA, SB, and sleep as being independent of each other [40, 54, 55]. A recent study analysed temporal trends in the prevalence of the Thai 24-hour integrated movement guidelines [56]. However, the CoDA approach has never been applied to analyse daily amounts of PA, SB, and sleep in Thailand, while statistically accounting for the co-dependency of these behaviours.

Evidence from several reviews has shown that younger age and higher education level are usually associated with higher PA among adults [57]. However, a recent review of 167 studies concluded that correlates of PA and SB may differ between countries and called for country-specific analyses of PA and SB correlates [58]. This review found male sex and younger age to be associated with higher PA only in children and adolescents, while age, sex, education level, household income, and marital status were not found to be associated with PA among adults and seniors [58].

The aim of the present study was, therefore, to determine the amounts of time spent in PA, SB, and sleep in the Thai population and across a range of its sociodemographic groups, using the two most recent national time-use surveys. We also aimed to determine relative changes in the time-use composition in the six-year period between the two surveys. We hypothesised that the amount of time spent in SB has increased at the expense of PA, while sleep remained constant. Based on previous evidence, we also hypothesised that sex, age, and education level would be important correlates of PA, SB, and sleep in the Thai population. This study may also serve as an example of how CoDA can be applied to analyse daily amounts of PA, SB, and sleep estimated from time-use surveys.

## Materials and methods

### Study design and participants

The study was based on cross-sectional data from the Thai National Time-Use Surveys conducted by the NSO in 2009 and 2015 [50, 51]. The data collections were conducted from July 2009 to September 2009 and from July 2014 to June 2015. The NSO used a three-stage stratified sampling design to generate a random sample of residential households from five regions (Bangkok, Central, North, North-East, and South) and 77 provinces with enumeration areas (municipal, non-municipal) [50, 51]. One individual aged 10 years or older in 2009 and 6 years or older in 2015 per household was invited to participate in the surveys. The NSO expanded the age range of participants in the latter survey to get insight into time use among children. However, to ensure that data from both waves are comparable, in the current study, we included only participants aged 10 years or older.

A total of 66,652 participants from the 2009 survey and 69,922 participants from the 2015 survey were included in the study. After excluding participants who reported no sleep (*n* = 367 in 2009 and *n* = 184 in 2015), no active wake time (*n* = 69 in 2009 and *n* = 80 in 2015), and no sedentary behaviour (*n* = 28 in 2009 and *n* = 22 in 2015), the final sample in the current study included 135,824 participants, including 66,188 participants from the 2009 survey and 69,636 from the 2015 survey.

The approval to conduct the surveys was given to the NSO by the Official Information Act, B.E. 2540 [59]. The survey was anonymous, and all participants provided informed consent before taking part in the survey. For all minors participating in these surveys, informed consent was obtained from their parents or guardians. For the purpose of the current study, permissions to access and use the data were obtained from the NSO. No data that would enable identifying individual participants before or after the data collection were available to the authors of this paper.

### Measures

The participants reported their time use over one day (24 hours) by recording activities that they performed in 10-minute intervals. For young children who could not report the time-use activities, the data were collected from proxy respondents (the parents or guardians). The time-use data were coded using the Trial ICATUS [60]. If a participant performed more than one activity at a time, they were instructed to record one of them as the main (primary) activity and the other one as a secondary activity. The participants were also asked to provide more information about place/location or where an activity occurred (e.g., at home, workplace, or school) for each 10-minute block.

The Trial ICATUS activity codes were classified into PA, SB, and sleep, according to previously developed criteria [44]. For work- and travel-related activities we used further data on occupation and location provided by the participants and linked them with the metabolic equivalent of the tasks that were already assigned to standard occupations and modes of travel in previous studies [42, 56, 61]. The PA category included PA of any intensity. The sleep category encompassed any sleep occurring in the 24-hour period, including overnight sleep and naps. Once all activities were categorised into PA, SB, and sleep, the total time for each category was computed. The time spent in these three behaviours summed up to 24 hours.

The NSO used a separate questionnaire to collect information on sociodemographic characteristics of the participants in the Thai National time-use surveys. The current study included sex (male and female), household area (urban and rural), age (adolescents, youth, young adults, middle-aged adults, youngest-older adults, middle-older adults, and oldest-older adults), education level (primary and below, secondary and high school, college, university and higher), employment status (employed and unemployed), marital status (never married, currently married, and formerly married), region (Bangkok, Central, North, North-East, and South), and day type (weekday and weekend day).

### Data analysis

We applied population weights given by the NSO and additional weights to achieve a uniform distribution of the days of the week in the analysis. We calculated compositional means for the overall sample by adjusting the geometric means of all parts of the time-use composition to collectively add up to 24 hours, and we presented them and their 95% confidence boundaries in a ternary plot. Proportionality of time-use components was expressed using the variation matrix.

To explore how the compositional parts (PA, SB and sleep) differed across the sociodemographic variables, the three parts of time-use composition were first expressed using two isometric log-ratio (*ilr*) coordinates. Two multiple regression analyses were then conducted for each survey year, with the *ilr*-transformed data as dependent variables and the sociodemographic variables as predictors. To obtain the least-squares mean for each sociodemographic category in each survey year, we used the unstandardised regression coefficients from the multivariate models. We then applied the inverse *ilr* transformation to determine the proportions of time spent in each compositional part. These proportions were linearly adjusted to collectively add up to 24 hours, and bootstrap 95% confidence intervals were generated for the obtained composition with 1000 replicates.

To test whether the change in the time-use compositions from 2009 to 2015 differed significantly between categories of a sociodemographic variable, the p-value for the interaction between the sociodemographic variable and survey year was calculated. Furthermore, to facilitate the interpretation of results, for each behaviour the difference in min/d between the two time points and its bootstrap 95% confidence interval were calculated. The calculations were done based on the compositional mean for the whole sample and least-squares means for sociodemographic categories.

All analyses were performed in R (R Foundation for Statistical Computing, Vienna, Austria), using ‘compositions’ [62], ‘zCompositions’ [63], ‘ggplot2’ [64], ‘ggtern’ [65], ‘dplyr’ [66], ‘boot’ [67], ‘car’ [68], ‘emmeans’ [69], ‘questionr’ [70] and ‘Rmpfr’ [71] packages.

## Results

### Sample characteristics

The sex distribution was similar in both survey years (*p* = 0.332) (Table 1). Almost 15% of people moved from rural to urban area during the study period (*p* < 0.001). The proportion of younger age groups (i.e., adolescents, youth, and young adults) declined, while that of other age groups (i.e., middle-aged adults, youngest-older adults, middle-older adults, and oldest-older adults) increased (*p* < 0.001). More people had a higher level of ***education*** in 2015, compared with 2009 (*p* < 0.001). However, in the same period the employment rate dropped (*p* < 0.001).

**Table 1:** Time (h/day) spent in physical activity, sedentary behaviour and sleep in the Thai population.

Category	*n (*%)		Physical activity		Sedentary behaviour		Sleep		*p*
			*ĝ* (95% CI)		*ĝ* (95% CI)		*ĝ* (95% CI)		
	2009	2015	2009	2015	2009	2015	2009	2015	
**Sex**									
*Female*	33877 (51.2)	35826 (51.4)	10.35 (10.27–10.43)	9.42 (9.36–9.48)	4.82 (4.76–4.89)	5.52 (5.47–5.56)	8.83 (8.78–8.88)	9.06 (9.03–9.10)	<0.001
*Male*	32311 (48.8)	33810 (48.6)	9.09 (8.99–9.18)	8.17 (8.10–8.25)	5.56 (5.50–5.63)	6.23 (6.17–6.28)	9.36 (9.30–9.41)	9.60 (9.56–9.64)	
**Household area**									
*Rural*	45573 (68.9)	38341 (55.1)	9.67 (9.59–9.75)	8.74 (8.67–8.80)	5.23 (5.17–5.29)	5.89 (5.84–5.94)	9.11 (9.06–9.15)	9.37 (9.34–9.41)	0.035
*Urban*	20615 (31.1)	31295 (44.9)	9.84 (9.76–9.93)	8.89 (8.82–8.96)	5.08 (5.01–5.14)	5.82 (5.77–5.87)	9.08 (9.03–9.13)	9.29 (9.25–9.32)	
**Age**									
*Adolescents (10–17 years)*	9303 (14.1)	8380 (12.0)	11.44 (11.14–11.76)	10.55 (10.29–10.83)	4.30 (4.09–4.50)	5.02 (4.84–5.18)	8.26 (8.10–8.43)	8.43 (8.28–8.58)	<0.001
*Youth (18–24 years)*	8146 (12.3)	7959 (11.4)	10.11 (9.85–10.37)	9.65 (9.48–9.81)	4.80 (4.64–4.99)	5.22 (5.10–5.35)	9.09 (8.95–9.24)	9.13 (9.03–9.23)	
*Young adults (25–39 years)*	18606 (28.1)	17670 (25.4)	9.99 (9.89–10.10)	9.14 (9.05–9.24)	4.92 (4.84–5.00)	5.55 (5.47–5.63)	9.09 (9.03–9.15)	9.31 (9.26–9.36)	
*Middle-aged adults (40–59 years)*	21367 (32.3)	23706 (34.0)	9.72 (9.62–9.81)	8.77 (8.70–8.85)	5.36 (5.29–5.43)	5.99 (5.94–6.05)	8.93 (8.87–8.98)	9.23 (9.19–9.28)	
*Youngest-older adults (60–74 years)*	6491 (9.8)	8940 (12.8)	8.12 (7.96–8.27)	7.45 (7.33–7.57)	6.22 (6.10–6.34)	6.78 (6.69–6.87)	9.66 (9.58–9.74)	9.77 (9.71–9.83)	
*Middle-older adults (75–84 years)*	1832 (2.8)	2409 (3.5)	5.27 (5.01–5.52)	5.36 (5.19–5.54)	7.71 (7.50–7.91)	7.79 (7.65–7.93)	11.03 (10.85–11.21)	10.85 (10.74–10.96)	
*Oldest-older adults (≥85 years)*	443 (0.7)	572 (0.8)	3.34 (2.99–3.73)	3.29 (3.02–3.57)	8.24 (7.86–8.60)	8.83 (8.53–9.13)	12.42 (12.13–12.72)	11.88 (11.67–12.10)	
**Education level**									
*Primary and below*	39893 (60.3)	35913 (51.6)	9.52 (9.43–9.61)	8.68 (8.61–8.76)	5.19 (5.12–5.26)	5.84 (5.79–5.90)	9.29 (9.23–9.34)	9.48 (9.43–9.52)	<0.001
*Secondary and high school*	17674 (26.7)	21366 (30.7)	10.10 (9.99–10.21)	9.02 (8.94–9.10)	5.05 (4.97–5.14)	5.72 (5.65–5.79)	8.85 (8.78–8.91)	9.26 (9.21–9.31)	
*College*, *university and higher*	8494 (12.8)	12073 (17.3)	9.89 (9.75–10.03)	8.78 (8.66–8.90)	5.37 (5.26–5.49)	6.20 (6.11–6.30)	8.74 (8.66–8.82)	9.02 (8.95–9.08)	
**Employment status**									
*Employed*	44451 (67.2)	44931 (64.5)	11.06 (10.97–11.15)	10.17 (10.11–10.23)	4.38 (4.31–4.44)	5.01 (4.96–5.05)	8.57 (8.52–8.61)	8.82 (8.79–8.86)	0.190
*Unemployed*	16290 (24.6)	20187 (29.0)	6.79 (6.66–6.93)	6.06 (5.98–6.15)	7.20 (7.07–7.31)	7.89 (7.81–7.97)	10.01 (9.94–10.09)	10.05 (10.00–10.10)	
**Marital status**									
*Never married*	14902 (22.5)	17350 (24.9)	9.11 (8.95–9.26)	8.25 (8.15–8.36)	5.62 (5.51–5.74)	6.27 (6.18–6.36)	9.27 (9.19–9.36)	9.48 (9.42–9.55)	<0.001
*Currently married*	39441 (59.6)	39197 (56.3)	10.00 (9.93–10.09)	9.07 (9.00–9.15)	5.02 (4.96–5.08)	5.71 (5.65–5.76)	8.98 (8.93–9.02)	9.22 (9.18–9.26)	
*Formerly married*	6401 (9.7)	8021 (11.5)	9.44 (9.26–9.62)	8.71 (8.56–8.84)	5.16 (5.03–5.29)	5.75 (5.65–5.84)	9.40 (9.29–9.50)	9.55 (9.47–9.63)	
**Region**									
*Bangkok*	6483 (9.8)	9141 (13.1)	9.93 (9.71–10.13)	8.89 (8.72–9.05)	5.20 (5.06–5.36)	5.70 (5.58–5.83)	8.87 (8.72–9.00)	9.41 (9.31–9.51)	<0.001
*Central*	15751 (23.8)	20115 (28.9)	9.84 (9.75–9.93)	8.95 (8.87–9.03)	5.03 (4.96–5.10)	5.83 (5.77–5.89)	9.13 (9.08–9.19)	9.23 (9.18–9.27)	
*North*	12344 (18.6)	12017 (17.3)	9.73 (9.63–9.85)	8.78 (8.69–8.87)	4.97 (4.89–5.05)	5.64 (5.58–5.71)	9.30 (9.23–9.36)	9.58 (9.53–9.63)	
*North-East*	22562 (34.1)	19174 (27.5)	9.67 (9.55–9.79)	8.67 (8.58–8.77)	5.26 (5.17–5.34)	6.02 (5.95–6.08)	9.08 (9.01–9.15)	9.31 (9.26–9.37)	
*South*	9048 (13.7)	9189 (13.2)	9.50 (9.37–9.63)	8.72 (8.61–8.83)	5.53 (5.42–5.63)	6.06 (5.97–6.15)	8.97 (8.90–9.05)	9.22 (9.16–9.28)	
**Type of day**									
*Weekday*	46415 (70.1)	49646 (71.3)	9.95 (9.88–10.03)	9.17 (9.11–9.23)	5.06 (5.01–5.11)	5.64 (5.60–5.68)	8.99 (8.95–9.03)	9.19 (9.16–9.22)	<0.001
*Weekend day*	19773 (29.9)	19990 (28.7)	9.20 (9.06–9.33)	7.92 (7.83–8.01)	5.45 (5.36–5.55)	6.41 (6.34–6.48)	9.35 (9.28–9.43)	9.66 (9.61–9.72)	

Notes: *ĝ* = weighted least-squares means adjusted to sum to 24 hours, calculated from compositional regression analyses with isometric log-ratio coordinates as outcome variables and all categories (sex, household area, age, education level, employment status, marital status, region, type of day) as explanatory variables; CI = bootstrap confidence interval for *ĝ*; *p* = p-value for the interaction between the explanatory variable and survey year.

### Time spent in physical activity, sedentary behaviour, and sleep

In 2009, the participants spent the largest amount of time in PA (compositional mean [*g*] = 9.50 h/day; 95% confidence interval [CI]: 9.43, 9.56). This was followed by the amount of time spent sleeping (*g* = 9.20 h/day; 95% CI: 9.16, 9.23), while the least time was spent in SB (*g* = 5.30 h/day; 95% CI: 5.26, 5.35). In 2015 this order changed, with the participants now spending most of their time sleeping (*g* = 9.44 h/day; 95% CI: 9.42, 9.47), followed by PA (*g* = 8.60 h/day; 95% CI: 8.55, 8.64) and SB (*g* = 5.96 h/day; 95% CI: 5.93, 6.00). The time spent in PA was found to be higher on weekdays, while the amounts of SB and sleep were found to be higher on weekends (*p* < 0.05). The *total variance*, calculated as the sum of the unique elements in the variation matrix, was 1.85 in both 2009 and 2015, while the proportionality between different parts of the composition also remained very similar between the survey years (S1 File).

Similar associations of the time-use composition with sociodemographic characteristics were found in both survey years (Table 1). Compared with their female counterparts, males spent a lower amount of time in PA and a higher amount of time in SB and sleep (*p* < 0.05 for all comparisons).

People living in the urban area had slightly higher PA and slightly lower SB, compared with people living in rural areas (*p* < 0.05 for both). We did not find a significant difference between the two groups in the amount of time spent sleeping.

The time spent in PA seemed to decrease by age, with those aged ≥85 years spending on average between 1.93 and 8.10 hours less time in PA than other age groups (*p* < 0.05). The amount of SB seemed to increase by age, with particularly large increases found for those aged ≥60 years. The three oldest age groups (≥60 years) also had the highest sleep duration.

The largest amount of PA was found among participants who finished secondary school and high school, while the largest amount of SB was found among those with college, university and higher degrees (*p* < 0.05). Participants who were least educated had the highest amount of sleep time (*p* < 0.05).

Those who were unemployed had a significantly lower amount of PA and significantly higher amounts of SB and sleep, compared with those who were employed (*p* < 0.05 for all). The average difference was around 4.25 hours for PA, 2.85 hours for SB, and almost 1.5 hours for sleep.

Participants who never got married had the lowest amount of PA and the highest amount of SB, while the currently married participants spent the largest amount of time in PA and lowest amount of time in sleep, compared with other groups by marital status (*p* < 0.05 for all comparisons).

We found no clear patterns in the time-use composition associated with geographical region.

### Changes in time-use composition over time

**Fig 1:**
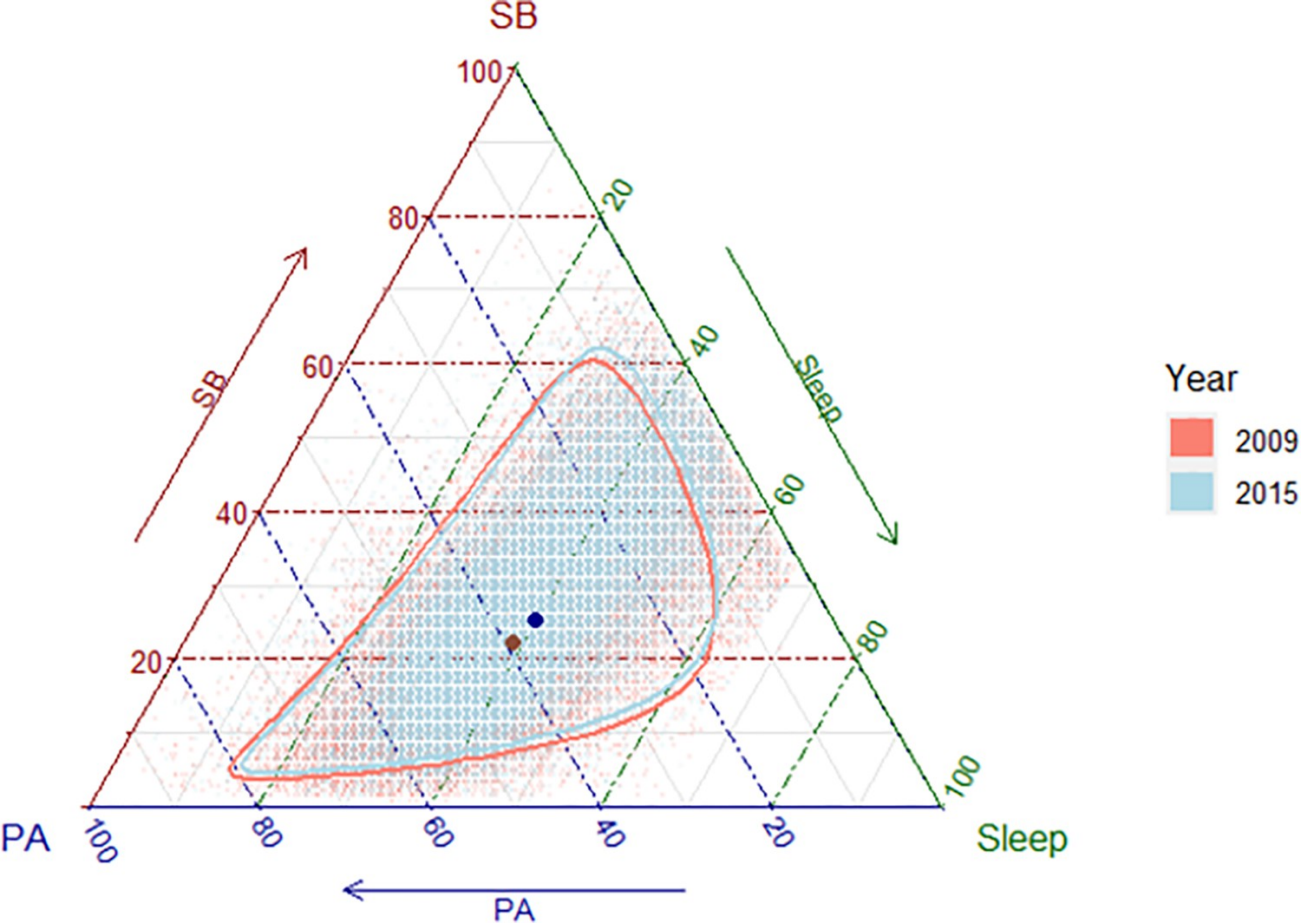
Compositional means of physical activity, sedentary behaviour, and sleep time in the Thai population in 2009 and 2015. The orange and blue circles on the graph represent the compositional means in 2009 and 2015, respectively. The orange and blue dots on the graph represent individual data points in 2009 and 2015, respectively. The orange and blue lines represent the 95% confidence boundaries for the compositional means in 2009 and in 2015, respectively. PA = physical activity; SB = sedentary behaviour.

**Fig 2:**
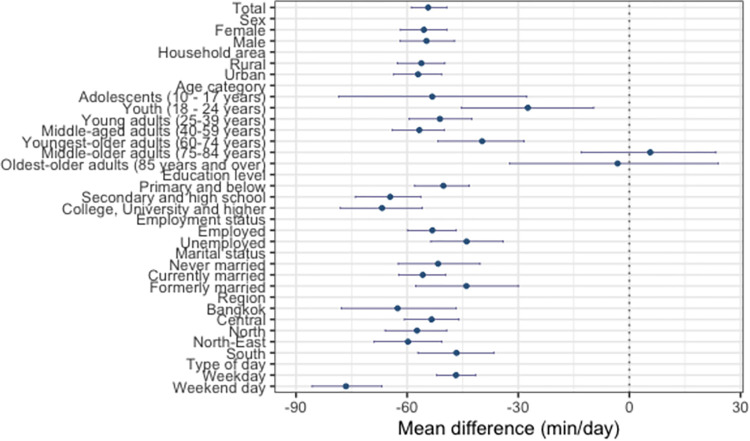
**Mean difference in physical activity from 2009 to 2015.** Mean changes in physical activity from 2009 to 2015 (circles) and their 95% bootstrap confidence intervals (whiskers).

**Fig 3:**
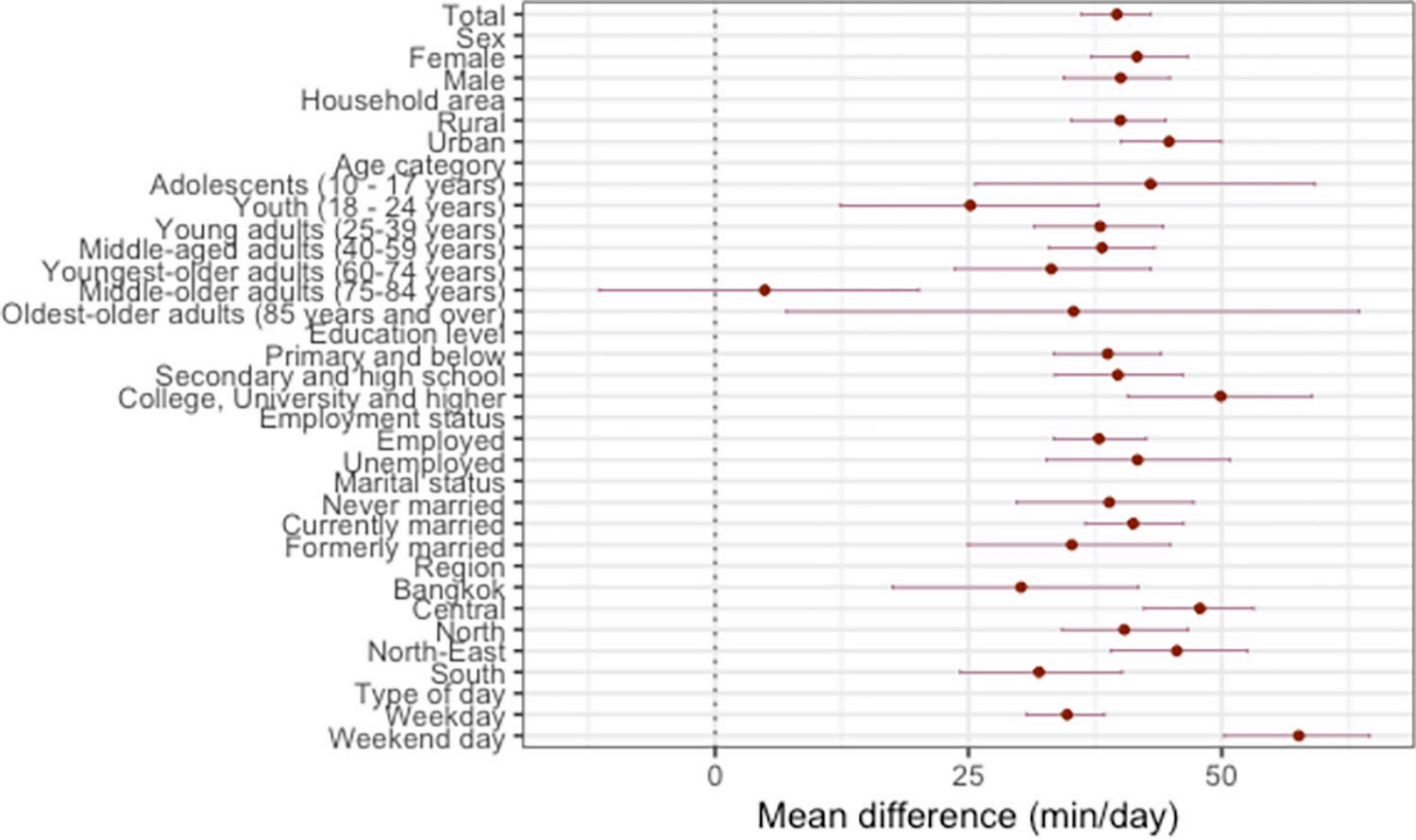
**Mean difference in sedentary behaviour from 2009 to 2015.** Mean changes in sedentary behaviour from 2009 to 2015 (circles) and their 95% bootstrap confidence intervals (whiskers).

**Fig 4:**
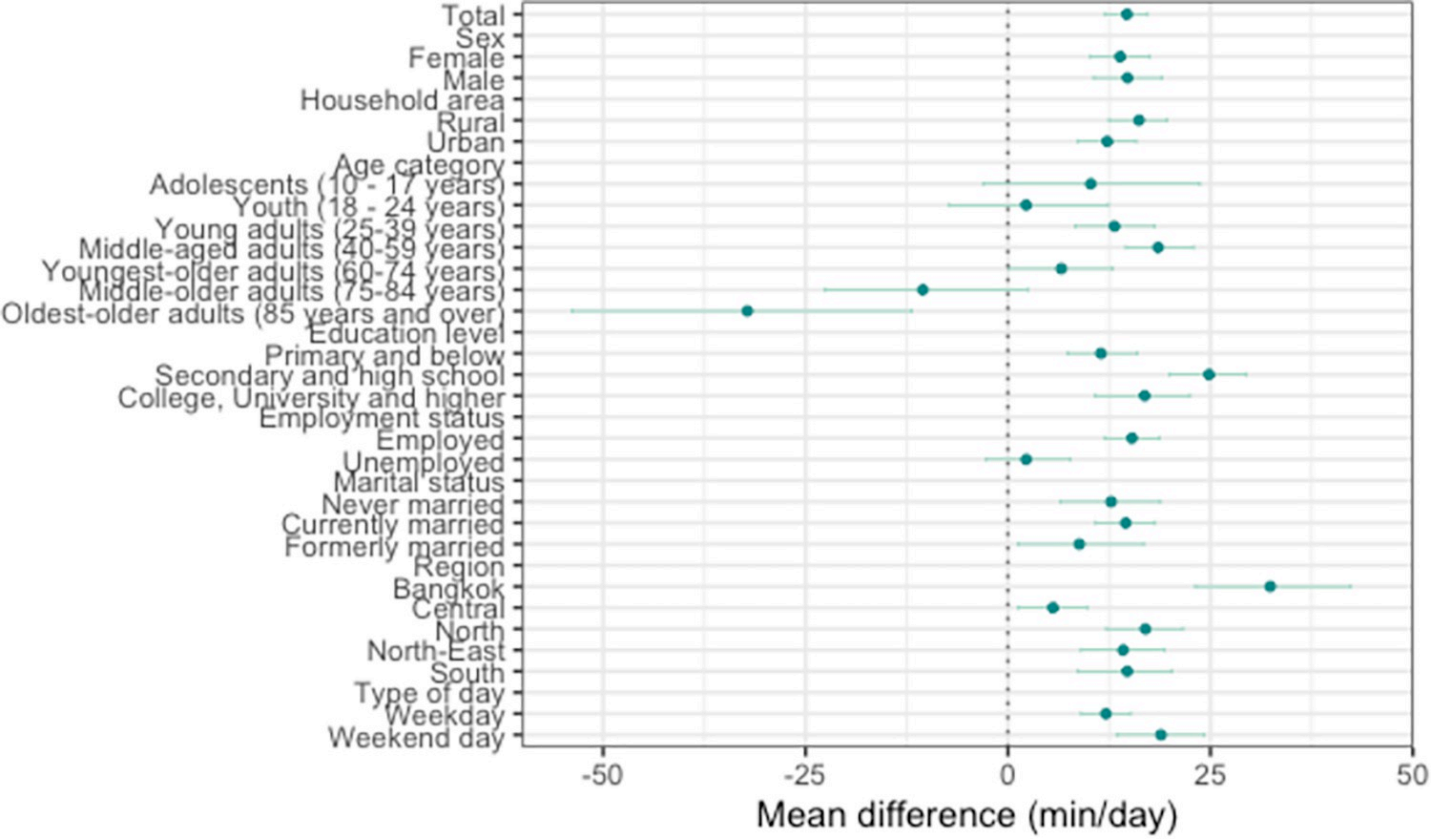
Mean difference in sleep duration from 2009 to 2015. Mean changes in sleep duration from 2009 to 2015 (circles) and their 95% bootstrap confidence intervals (whiskers).

The results show that SB and sleep increased at the expense of PA between 2009 and 2015 (Figs 1–4). On average, the time spent in PA decreased by 54.33 min/day (95% CI: -58.88, -49.30; Fig 2), with a significantly larger decrease in PA found on weekend days (mean difference [*d*] = -76.52 min/day; 95% [CI]: -85.68, -66.81), compared with weekdays (*d* = -46.82 min/day; 95% [CI]: -52.02, -41.46). The time spent in SB increased by 39.64 min/day (95% CI: 36.18, 42.98; Fig 3), with a significantly larger increase in SB found on weekend days (*d* = 57.61 min/day; 95% [CI]: 50.22, 64.63), compared with weekdays (*d* = 34.73 min/day; 95% [CI]: 30.68, 38.44). Sleep time increased by 14.69 min/day (95% CI: 11.89, 17.28; Fig 4), with no significant difference in the change over time between weekends and weekdays.

All sociodemographic characteristics, except for employment status, significantly moderated the changes in the overall time-use composition between 2009 and 2015 (*p* < 0.05 for all; Table 1). Those with college, university or higher education had the largest decrease in PA (*d* = -66.78 min/day; 95% CI: -78.00, -55.86) and the largest increase in SB (*d* = 49.90 min/day, 95% CI: 40.70, 58.87), compared with other groups (Figs 2 and 3). The oldest older adults were the only sociodemographic group for which we found a significant decrease in the amount of sleep between the two surveys (*d* = -32.19 min/day; 95% CI: -53.86, -11.84; Fig 4).

## Discussion

### Main findings

In accordance with our hypotheses, the main findings of this study are relatively large increases in SB and decreases in PA in the Thai population between 2009 and 2015. These findings were consistent across most sociodemographic groups, with the most concerning shifts from active to sedentary lifestyle found for those with a higher education degree. In both survey years we found that males, older age groups, and those who were unemployed tended to spend less time in PA and more time in SB, compared with other population groups. We also found that the amount of PA was higher on weekdays, while the amounts of SB and sleep were higher on weekends. Compared with PA and SB, sleep duration increased only slightly over time, with relatively consistent increases found on weekdays and weekends, and across most population groups.

### Time-use composition and its changes over time

We found that, on average, more than 40 minutes of PA was replaced by SB in the Thai population between 2009 and 2015, with little change in sleep duration. This is consistent with findings from international studies that show a shift from physically active to sedentary lifestyles [72, 73], and an upward trend of SB and physical inactivity in several countries around the world [74–77].

The 4^th^ and 5^th^ National Health Examination Survey (NHES) found that Thai people aged 15 years and older increased their moderate-to-vigorous PA, from an average of 1.4 h/day in 2009 to 2 h/day in 2014 [78, 79]. Given that we analysed overall PA (including light PA and moderate-to-vigorous PA), it may be that the decline in PA we found was mainly driven by a decline in light PA. Furthermore, the data from two Thailand’s Surveillance on Physical Activity surveys showed a fluctuating trend. They found that Thai adults (aged 18 years and over) engaged in moderate-to-vigorous PA on average for 11.7 h/week in 2012, 13.8 h/week in 2013, 11.2 h/week in 2014, and 9.1 h/week in 2015 [80]. If we only consider the first and the last year in their survey, our findings are in accordance with their results. It may be that the levels of PA in the Thai population are fluctuating over time, with an overall downwards trend.

The most recent Global Observatory for Physical Activity (GoPA!) survey found that the average time spent in SB ranges between 2.2 and 9.9 h/day across 164 countries [81]. It is concerning that the mean time spent in SB found for Thailand in the current study (for year 2015) was 1.3 h/day higher than the global average of 4.6 h/day from the GoPA! survey [81]. In Thailand and other low- and middle-income countries, research on SB is much less developed than PA research [40, 82]. Nevertheless, some data on SB in the Thai population are available from previous studies. The time spent in SB in the Thai population varied widely across these previous studies. A large-scale survey conducted in 2014 showed a high SB level of on average 13.4 h/day among Thais aged 5 years and over [83], while the 2015 Health and Welfare survey found Thai people aged 15 years and over spent on average only 1.9 h/day in SB [84]. Our estimate of SB was lower than in the former, and higher than in the latter study. It may be that these large differences are due to recall errors or how SB was defined in the questionnaires [85, 86].

The differences in findings on PA and SB between our study and previous Thai studies may also be partially explained by the fact that the previous studies considered PA, SB, and sleep durations as being independent of each other, while we used CoDA to acknowledge their belonging to a single time-use composition. The major difference between a CoDA approach and more conventional statistical techniques is in the treatment of time-use data. CoDA allows all parts of the time-use composition to be included in the same statistical model and it accounts for the relative and constrained nature of compositional data [15–17]. Prior studies have compared findings obtained from CoDA and traditional statistical approaches and found different estimates of time spent in different behaviours and their associations with health indicators [21, 29, 87]. One of the studies showed that estimates of differences in time spent in SB and PA at work were 15% larger between sexes, and 60% larger between age groups when using CoDA, compared to a “non-compositional” analysis [87].

### Time use on weekdays vs. weekend days

Our study found that the amount of PA was higher on weekdays, while the amounts of SB and sleep were higher on weekends. It is likely that most people have more free time on weekend days [88]. It seems that Thai people spend more of this additional free time on SB than on PA. This is consistent with findings of prior systematic reviews showing greater screen time (a common type of SB) among adolescents on weekends than on weekdays [89, 90]. Familial influence has been identified as a significant predictor of screen-based behaviours among all family members during weekend days [91, 92]. It may also be that schools and other organisational environments that provide physical education and exercise classes, contribute to school-aged adolescents being more active on weekdays than on weekends [93, 94].

While our results are in accordance with previous findings for adolescents, they are in contrast with some studies conducted among adults that found a higher amount of SB on weekdays than on weekend days [95, 96]. This may be because of the differences between the study samples. The samples in the two previous studies included working-age adults with a high education degree, so they potentially engaged in sedentary work, while our sample included also adolescents and older adults, and most participants did not have a college/university degree. The difference may also be due to cross-country differences in patterns and determinants of PA and SB [58].

A detailed exploration of reasons why Thai people are more sedentary and less physically active on weekends would be needed to inform future public health interventions. Based on our results we can only conclude that interventions should focus on increasing PA and reducing SB, particularly on weekend days. These interventions can focus on increasing recreational PA by enabling activity-friendly environments and equitable access to public open spaces in the community such as parks, playgrounds, and other facilities for sports and recreation. The quality of existing public spaces and facilities may need to be improved to ensure adequate accessibility and safety. A recent study on the association between park characteristics and PA in Thailand suggested that the quality of parks and better accessibility enabling equal opportunities for park use were important factors to promote park users to engage in more PA [97].

We also found that between 2009 and 2015 the decrease in PA and increase in SB was greater on weekend days, compared to weekdays. This further highlights the importance of placing a focus on weekend days when designing public health interventions to promote healthy time use.

### Population groups at the highest risk of unhealthy and deteriorating time use

According to our findings, between 2009 and 2015 the amount of time spent in PA decreased by more than one hour among those with a higher education degree. This unfavourable change is additionally concerning because of a fast increase in the number of highly educated people in Thailand. This population group is likely to continue growing in the future, and it should, therefore, be given a special consideration in national health strategies. Public health interventions should aim to prevent further decline in PA and increase in SB among those with a higher education degree. This may include, but should not be limited to, workplace PA promotion programs. As those with higher education are generally more likely to be office-based workers who spend a substantial amount of their time sitting at work, workplace programs to break and reduce sitting time should be supported. A recent survey of PA promotion in four settings (schools, workplaces, health facilities, and local administrative organizations) showed that only 27% of workplaces in Thailand had policies and environments (i.e., infrastructure, equipment, and “healthy” space) conducive to PA [98]. More workplaces should, therefore, implement programs and PA-friendly designs to increase opportunities for incidental movement throughout the workday for their employees. This may be achieved by facilitating the use of stairways and walking-friendly routes inside and outside buildings and by providing sit-stand desks and standing-up areas in meeting/conference rooms [99, 100].

The other Thai population groups that seem to be at risk of an unfavourable composition of PA and SB are males, older age groups, and unemployed people. In these groups PA levels were already low in 2009 and then further declined in 2015, albeit seemingly less than among those with a higher education degree. These population groups should be specifically targeted by public health interventions that aim to increase PA and reduce SB. Appropriately-designed programs and services to support older adults and other disadvantaged groups such as unemployed people to have equal opportunities for PA should be promoted. For example, this can be facilitated by village health volunteers who play an important role in leading health and quality of life promotion programs for people in the Thai communities. Such volunteers can reach out to the least physically active groups and help them increase their health awareness and participation in PA. A whole of community approach involving key stakeholders from different sectors (e.g. local governments, primary care units, and private businesses) is required to maximise opportunities for increased participation in PA for the least physically active groups [101].

Furthermore, older adults often experience fragmented sleep [102], and the amount of deep sleep seems to reduce in this age group, especially after the age of 90 years [103]. These changes may easily lead to more time spent lying in bed or in other sedentary activities. Although between two survey years we found a decrease in the amount of sleep in favour of SB for the oldest-older adults, this is unlikely to be concerning, because their sleep duration in 2009 was relatively high.

### Implications for public health

The replacement of approximately 40 minutes of PA by SB in the Thai population in the period between 2009 and 2015 is likely to have a negative effect on a range of health outcomes. According to several prior investigations, reallocating time from moderate-to-vigorous PA to SB is significantly associated with adverse health outcomes among all age groups [19, 21, 25, 30–32, 35, 104]. Replacing only 10 min of moderate-to-vigorous PA with SB has been associated with increased body mass index (BMI) of 5.1% in Canadian children and adolescents [19]. In US adults, an increase in BMI of 1.2% was associated with a 10-minute time replacement of moderate-to-vigorous PA by SB [21]. A 15-minute reduction of moderate-to-vigorous PA in favour of any of the other movement behaviours was also associated with higher cardio-metabolic risk in Australian older adults [25] and with a higher mortality risk in American adults [35]. Moreover, reallocation of 30 minutes away from moderate-to-vigorous PA to any of the other behaviours was associated with lower insulin sensitivity among adults in the United Kingdom [104]. A recent review of isotemporal substitution studies concluded that replacing PA (including light PA and moderate-to-vigorous PA) with SB is associated with increased mortality risk and unfavourable changes in weight status and cardio-metabolic markers [3]. Key implication of these findings is that maintaining or increasing population level of PA over time is crucial and should be advocated as a primary strategic goal in future public health interventions in Thailand.

### Strengths and limitations of the study

Methodological strengths of our study are: (1) a large, nationally representative sample, covering a broad age range; (2) the application of the widely-used ICATUS categorisation of activities and standardised method for their re-classification into PA, SB, and sleep [44], which may ensure good comparability with future studies on this topic based on ICATUS data; (3) the use of CoDA approach that enabled us to adequately address compositional properties of time-use data; and (4) the exploration of time-use patterns in a total of 24 population groups.

Key limitations of our study stem from the use of time-use survey data. First, as it is the case with other self-reports on PA, SB, and sleep, time-use surveys may be susceptible to recall bias. Second, it is possible that we missed activities that were performed for less than 10 consecutive minutes, as they would not be reported according to the ICATUS guidelines [60]. Third, the participants reported activities performed on a single day, which may have reduced reliability of estimates. Nevertheless, it should be noted that these are standard procedures for collecting time-use data, and they have shown to yield estimates with sufficient reliability and validity for observational studies [48]. Fourth, we did not assess possible interaction effects. Given the large number of possible interactions between explanatory variables in our regression model (*n* = 28), such analysis was beyond the scope of our study.

## Conclusions

Our findings revealed a shift to a more sedentary lifestyle in the Thai population, as a significant amount of PA was replaced by SB over time. The fastest deteriorating use of time was found among those with a higher education degree, while the most unfavourable time-use composition was found among males, older age groups, and those who are unemployed. The time use was generally less favourable on weekend days than on weekdays.

Given that the engagement in SB is increasing and has become a significant part of people’s lifestyles, immediate actions to prevent its likely negative impacts on population health are needed. Public health interventions should aim to prevent the rapid decrease in PA and increase in SB among those with a higher education degree such as implementing workplace PA promotion programs. They should also focus on increasing PA and reducing SB, particularly among the population groups whose time-use composition is already unfavourable. Future intervention programs should be designed to improve time use on weekends.

To further contribute to time-use epidemiology, more studies analysing time-use survey data using CoDA are needed.

## Supporting information

S1 File Variation matrix for the time-use composition. (PDF)

## References

[1] Rollo S, Antsygina O, Tremblay MS. The whole day matters: Understanding 24-hour movement guideline adherence and relationships with health indicators across the lifespan. Journal of Sport and Health Science. 2020;9(6):493–510. doi: 10.1016/j.jshs.2020.07.004 32711156 PMC7749249

[2] Janssen I, Clarke AE, Carson V, Chaput J-P, Giangregorio LM, Kho ME, et al. A systematic review of compositional data analysis studies examining associations between sleep, sedentary behaviour, and physical activity with health outcomes in adults. Applied Physiology, Nutrition, and Metabolism. 2020;45(10):S248–S57. doi: 10.1139/apnm-2020-0160 33054342

[3] Grgic J, Dumuid D, Bengoechea EG, Shrestha N, Bauman A, Olds T, et al. Health outcomes associated with reallocations of time between sleep, sedentary behaviour, and physical activity: a systematic scoping review of isotemporal substitution studies. International Journal of Behavioral Nutrition and Physical Activity. 2018;15(1):69. doi: 10.1186/s12966-018-0691-3 30001713 PMC6043964

[4] Posadzki P, Pieper D, Bajpai R, Makaruk H, Könsgen N, Neuhaus AL, et al. Exercise/physical activity and health outcomes: an overview of Cochrane systematic reviews. BMC Public Health. 2020;20(1):1724. doi: 10.1186/s12889-020-09855-3 33198717 PMC7670795

[5] Warburton DER, Bredin SSD. Health benefits of physical activity: a systematic review of current systematic reviews. 2017;32(5):541–56. doi: 10.1097/HCO.0000000000000437 28708630

[6] Jakicic JM, Kraus WE, Powell KE, Campbell WW, Janz KF, Troiano RP, et al. Association between Bout Duration of Physical Activity and Health: Systematic Review. Medicine and science in sports and exercise. 2019;51(6):1213–9. doi: 10.1249/MSS.0000000000001933 31095078 PMC6527142

[7] Saunders TJ, McIsaac T, Douillette K, Gaulton N, Hunter S, Rhodes RE, et al. Sedentary behaviour and health in adults: an overview of systematic reviews. 2020;45(10 (Suppl. 2)):S197–S217. doi: 10.1139/apnm-2020-0272 33054341

[8] Proper KI, Singh AS, Van Mechelen W, Chinapaw MJ. Sedentary behaviors and health outcomes among adults: a systematic review of prospective studies. American Journal of Preventive Medicine. 2011;40(2):174–82. doi: 10.1016/j.amepre.2010.10.015 21238866

[9] Biswas A, Oh PI, Faulkner GE, Bajaj RR, Silver MA, Mitchell MS, et al. Sedentary time and its association with risk for disease incidence, mortality, and hospitalization in adults: a systematic review and meta-analysis. Annals of internal medicine. 2015;162(2):123–32. doi: 10.7326/M14-1651 25599350

[10] Dunstan DW, Howard B, Healy GN, Owen N. Too much sitting–a health hazard. Diabetes research and clinical practice. 2012;97(3):368–76. doi: 10.1016/j.diabres.2012.05.020 22682948

[11] Owen N, Healy GN, Matthews CE, Dunstan DW. Too much sitting: the population-health science of sedentary behavior. Exercise and sport sciences reviews. 2010;38(3):105–13. doi: 10.1097/JES.0b013e3181e373a2 20577058 PMC3404815

[12] Chattu VK, Manzar MD, Kumary S, Burman D, Spence DW, Pandi-Perumal SR. The Global Problem of Insufficient Sleep and Its Serious Public Health Implications. Healthcare (Basel). 2018;7(1):1. doi: 10.3390/healthcare7010001 30577441 PMC6473877

[13] Itani O, Jike M, Watanabe N, Kaneita Y. Short sleep duration and health outcomes: a systematic review, meta-analysis, and meta-regression. Sleep Medicine. 2017;32:246–56. doi: 10.1016/j.sleep.2016.08.006 27743803

[14] Chastin SF, Palarea-Albaladejo J. Concise Guide to Compositional Data Analysis for Physical Activity, Sedentary Behavior, and Sleep Research: Supplementary Material S2, in Chastin SFM, Palarea-Albaladejo J, Dontje ML, Skelton DA. "Combined effects of time spent in physical activity, sedentary behaviors and sleep on obesity and cardio-metabolic health markers: a novel compositional data analysis approach". PloS one. 2015;10(10):e0139984. 26461112 10.1371/journal.pone.0139984 PMC4604082

[15] Dumuid D, Stanford TE, Martin-Fernández J-A, Pedišić Ž, Maher CA, Lewis LK, et al. Compositional data analysis for physical activity, sedentary time and sleep research. Statistical methods in medical research. 2018;27(12):3726–38. doi: 10.1177/0962280217710835 28555522

[16] Pedišić Ž, Dumuid D, S Olds T. Integrating sleep, sedentary behaviour, and physical activity research in the emerging field of time-use epidemiology: definitions, concepts, statistical methods, theoretical framework, and future directions. Kinesiology: International journal of fundamental and applied kinesiology. 2017;49(2):252–69.

[17] Dumuid D, Pedišić Ž, Palarea-Albaladejo J, Martín-Fernández JA, Hron K, Olds T. Compositional data analysis in time-use epidemiology: what, why, how. International journal of environmental research and public health. 172020. p. 2220. doi: 10.3390/ijerph17072220 32224966 PMC7177981

[18] Trinh HT, Morais J, Thomas-Agnan C, Simioni M. Relations between socio-economic factors and nutritional diet in Vietnam from 2004 to 2014: New insights using compositional data analysis. Stat Methods Med Res. 2019;28(8):2305–25. doi: 10.1177/0962280218770223 29683048

[19] Carson V, Tremblay MS, Chaput J-P, Chastin SF. Associations between sleep duration, sedentary time, physical activity, and health indicators among Canadian children and youth using compositional analyses. Applied Physiology, Nutrition, and Metabolism. 2016;41(6):S294–S302. doi: 10.1139/apnm-2016-0026 27306435

[20] Carson V, Tremblay MS, Chastin SFM. Cross-sectional associations between sleep duration, sedentary time, physical activity, and adiposity indicators among Canadian preschool-aged children using compositional analyses. BMC Public Health. 2017;17(5):848. doi: 10.1186/s12889-017-4852-0 29219077 PMC5773862

[21] Chastin SF, Palarea-Albaladejo J, Dontje ML, Skelton DA. Combined effects of time spent in physical activity, sedentary behaviors and sleep on obesity and cardio-metabolic health markers: a novel compositional data analysis approach. PloS one. 2015;10(10):e0139984. doi: 10.1371/journal.pone.0139984 26461112 PMC4604082

[22] McGregor D, Carson V, Palarea-Albaladejo J, Dall P, Tremblay M, Chastin S. Compositional analysis of the associations between 24-h movement behaviours and health indicators among adults and older adults from the canadian health measure survey. International journal of environmental research and public health. 2018;15(8):1779. doi: 10.3390/ijerph15081779 30126215 PMC6121426

[23] McGregor D, Palarea-Albaladejo J, Dall P, Stamatakis E, Chastin S. Differences in physical activity time-use composition associated with cardiometabolic risks. Preventive medicine reports. 2019;13:23–9. doi: 10.1016/j.pmedr.2018.11.006 30456055 PMC6240623

[24] Rodríguez-Gómez I, Mañas A, Losa-Reyna J, Rodríguez-Mañas L, Chastin SF, Alegre LM, et al. Associations between sedentary time, physical activity and bone health among older people using compositional data analysis. PloS one. 2018;13(10):e0206013. doi: 10.1371/journal.pone.0206013 30346973 PMC6197664

[25] Dumuid D, Lewis L, Olds T, Maher C, Bondarenko C, Norton L. Relationships between older adults’ use of time and cardio-respiratory fitness, obesity and cardio-metabolic risk: A compositional isotemporal substitution analysis. Maturitas. 2018;110:104–10. doi: 10.1016/j.maturitas.2018.02.003 29563028

[26] Dumuid D, Maher C, Lewis LK, Stanford TE, Fernández JAM, Ratcliffe J, et al. Human development index, children’s health-related quality of life and movement behaviors: A compositional data analysis. Quality of Life Research. 2018;27:1473–82. doi: 10.1007/s11136-018-1791-x 29362939 PMC7484943

[27] Dumuid D, Olds T, Lewis LK, Martin-Fernández JA, Katzmarzyk PT, Barreira T, et al. Health-related quality of life and lifestyle behavior clusters in school-aged children from 12 countries. The Journal of pediatrics. 2017;183:178–83. e2. doi: 10.1016/j.jpeds.2016.12.048 28081885

[28] Dumuid D, Pedišić Ž, Stanford TE, Martín-Fernández J-A, Hron K, Maher CA, et al. The compositional isotemporal substitution model: A method for estimating changes in a health outcome for reallocation of time between sleep, physical activity and sedentary behaviour. Statistical methods in medical research. 2019;28(3):846–57. doi: 10.1177/0962280217737805 29157152

[29] Dumuid D, Stanford TE, Pedišić Ž, Maher C, Lewis LK, Martín-Fernández J-A, et al. Adiposity and the isotemporal substitution of physical activity, sedentary time and sleep among school-aged children: a compositional data analysis approach. BMC Public Health. 2018;18(1):311. doi: 10.1186/s12889-018-5207-1 29499689 PMC5834855

[30] Dumuid D, Wake M, Clifford S, Burgner D, Carlin JB, Mensah FK, et al. The Association of the Body Composition of Children with 24-Hour Activity Composition. The Journal of pediatrics. 2019;208:43–9. doi: 10.1016/j.jpeds.2018.12.030 30704791

[31] Fairclough SJ, Dumuid D, Mackintosh KA, Stone G, Dagger R, Stratton G, et al. Adiposity, fitness, health-related quality of life and the reallocation of time between children’s school day activity behaviours: A compositional data analysis. Preventive medicine reports. 2018;11:254–61. doi: 10.1016/j.pmedr.2018.07.011 30109170 PMC6080199

[32] Fairclough SJ, Dumuid D, Taylor S, Curry W, McGrane B, Stratton G, et al. Fitness, fatness and the reallocation of time between children’s daily movement behaviours: an analysis of compositional data. international journal of behavioral nutrition and physical activity. 2017;14(1):64. doi: 10.1186/s12966-017-0521-z 28486972 PMC5424384

[33] Gupta N, Korshøj M, Dumuid D, Coenen P, Allesøe K, Holtermann A. Daily domain-specific time-use composition of physical behaviors and blood pressure. International Journal of Behavioral Nutrition and Physical Activity. 2019;16(1):4. doi: 10.1186/s12966-018-0766-1 30630517 PMC6327498

[34] von Rosen P, Dohrn M, Hagströmer M. Association between physical activity and all‐cause mortality: a 15‐year follow‐up using a compositional data analysis. Scandinavian Journal of Medicine & Science in Sports. 2019. doi: 10.1111/sms.13561 31581345

[35] Clarke AE, Janssen I. A compositional analysis of time spent in sleep, sedentary behaviour and physical activity with all-cause mortality risk. International Journal of Behavioral Nutrition and Physical Activity. 2021;18(1):1–12. 33549100 10.1186/s12966-021-01092-0 PMC7866642

[36] Kitano N, Kai Y, Jindo T, Tsunoda K, Arao T. Compositional data analysis of 24-hour movement behaviors and mental health in workers. Preventive medicine reports. 2020;20:101213. doi: 10.1016/j.pmedr.2020.101213 33204604 PMC7648171

[37] Pedišić Ž, Zhong A, Hardy LL, Salmon J, Okely ADOD, Chau J, et al. Physical activity prevalence in Australian children and adolescents: why do different surveys provide so different estimates, and what can we do about it? Kinesiology. 2017;49(2):135–45.

[38] Pedišić Ž, Bauman A. Accelerometer-based measures in physical activity surveillance: current practices and issues. Br J Sports Med. 2015;49(4):219–23. doi: 10.1136/bjsports-2013-093407 25370153

[39] Shephard RJ, Aoyagi Y. Measurement of human energy expenditure, with particular reference to field studies: an historical perspective. European journal of applied physiology. 2012;112(8):2785–815. doi: 10.1007/s00421-011-2268-6 22160180

[40] Liangruenrom N, Suttikasem K, Craike M, Bennie JA, Biddle SJH, Pedisic Z. Physical activity and sedentary behaviour research in Thailand: a systematic scoping review. BMC Public Health. 2018;18(1):733. doi: 10.1186/s12889-018-5643-y 29898706 PMC6001063

[41] United Nations Statistics Division. Gender Statistics: Department of Economic and Social Affairs, United Nations; 2018 [Available from: https://unstats.un.org/unsd/gender/timeuse/].

[42] Tudor-Locke C, Washington TL, Ainsworth BE, Troiano RP. Linking the American Time Use Survey (ATUS) and the compendium of physical activities: methods and rationale. Journal of Physical Activity and Health. 2009;6(3):347–53. doi: 10.1123/jpah.6.3.347 19564664

[43] Chau JY, Merom D, Grunseit A, Rissel C, Bauman AE, van der Ploeg HP. Temporal trends in non-occupational sedentary behaviours from Australian Time Use Surveys 1992, 1997 and 2006. International Journal of Behavioral Nutrition and Physical Activity. 2012;9(1):76. doi: 10.1186/1479-5868-9-76 22713740 PMC3419123

[44] Liangruenrom N, Craike M, Dumuid D, Biddle S, Tudor-Locke C, Ainsworth BE, et al. Standardised criteria for classifying the International Classification of Activities for Time-Use Statistics (ICATUS) activity groups into sleep, sedentary behaviour, and physical activity. International Journal of Behavioral Nutrition and Physical Activity. 2019;16:106. doi: 10.1186/s12966-019-0875-5 31727080 PMC6857154

[45] van Tienoven TP, Deyaert J, Harms T, Weenas D, Minnen J, Glorieux I. Active work, passive leisure? Associations between occupational and non-occupational physical activity on weekdays. Social science research. 2018;76:1–11. doi: 10.1016/j.ssresearch.2018.08.012 30268271

[46] Spinney JE, Millward H, Scott DM. Measuring active living in Canada: A time-use perspective. Social Science Research. 2011;40(2):685–94.

[47] Espinel PT, Chau JY, van der Ploeg HP, Merom D. Older adults’ time in sedentary, light and moderate intensity activities and correlates: application of Australian time use survey. Journal of science and medicine in sport. 2015;18(2):161–6. doi: 10.1016/j.jsams.2014.02.012 24702944

[48] van der Ploeg HP, Merom D, Chau JY, Bittman M, Trost SG, Bauman AE. Advances in population surveillance for physical activity and sedentary behavior: reliability and validity of time use surveys. American Journal of Epidemiology. 2010;172(10):1199–206. doi: 10.1093/aje/kwq265 20855469

[49] Bauman A, Bittman M, Gershuny J. A short history of time use research; implications for public health. BMC Public Health. 2019;19(2):607. 31159790 10.1186/s12889-019-6760-y PMC6546621

[50] National Statistical Office. The Time Use Survey 2015. Bangkok, Thailand: National Statistical Office, Ministry of Information and Communication Technology; 2016. Report No.: ISBN 978-974-11-3056-6.

[51] National Statistical Office. The Time Use Survey 2009. Bangkok, Thailand: National Statistical Office, Ministry of Information and Communication Technology; 2011. Report No.: ISBN 978-974-11-3056-6.

[52] National Statistical Office. The Time Use Survey 2001. Bangkok: National Statistical Office, Ministry of Information and Communication Technology; 2002.

[53] National Statistical Office. The Time Use Survey 2004. Bangko, Thailand: National Statistical Office, Ministry of Information and Communication Technology; 2005.

[54] Chaiard J, Deeluea J, Suksatit B, Songkham W, Inta N. Short sleep duration among Thai nurses: Influences on fatigue, daytime sleepiness, and occupational errors. Journal of occupational health. 2018;60(5):348–55. doi: 10.1539/joh.2017-0258-OA 29743391 PMC6176030

[55] Yiengprugsawan V, Banwell C, Seubsman S-a, Sleigh AC, Team TCS. Short sleep and obesity in a large national cohort of Thai adults. BMJ open. 2012;2(1):e000561. 10.1136/bmjopen-2011-000561 PMC3274710 22307100

[56] Liangruenrom N, Dumuid D, Craike M, Biddle SJ, Pedisic Z. Trends and correlates of meeting 24-hour movement guidelines: a 15-year study among 167,577 Thai adults. International Journal of Behavioral Nutrition and Physical Activity. 2020;17(1):1–17. 32838796 10.1186/s12966-020-01011-9 PMC7446156

[57] Bauman A, Reis RS, Sallis JF, Wells JC, Loos RJ, Martin BW, et al. Correlates of physical activity: why are some people physically active and others not? The lancet. 2012;380(9838):258–71. doi: 10.1016/S0140-6736(12)60735-1 22818938

[58] Liangruenrom N, Craike M, Biddle SJH, Suttikasem K, Pedisic Z. Correlates of physical activity and sedentary behaviour in the Thai population: a systematic review. BMC Public Health. 2019;19(1):414. doi: 10.1186/s12889-019-6708-2 30991973 PMC6469108

[59] National Statistical Office. About the National Statistical Office Bangkok: National Statistical Office; 2004 [Available from: http://web.nso.go.th/en/abt.htm].

[60] United Nations Statistics Division (UNSD). Guide to producing statistics on time use: Measuring paid and unpaid work. New York: Department of Economic and Social Affairs, United Nations; 2005.

[61] Deyaert J, Harms T, Weenas D, Gershuny J, Glorieux I. Attaching metabolic expenditures to standard occupational classification systems: perspectives from time-use research. BMC public health. 2017;17(1):620. doi: 10.1186/s12889-017-4546-7 28673271 PMC5496391

[62] van den Boogaart KG, Tolosana R, Bren M. Package ‘compositions’ Compositional Data Analysis. 2021.

[63] Palarea-Albaladejo J, Martin-Fernandez JA, Palarea-Albaladejo MJ. Package ‘zCompositions’ Treatment of Zeros, Left-Censored and Missing Values in Compositional Data Sets. 2020.

[64] Wickham H, Chang W, Wickham MH. Package ‘ggplot2’ Create Elegant Data Visualisations Using the Grammar of Graphics. 2016. p. 1–189.

[65] Hamilton N. Package ‘ggtern’ An Extension to ’ggplot2’, for the Creation of Ternary Diagrams. 2020.

[66] Wickham H, François R, Henry L, Müller K. dplyr: a grammar of data manipulation. R package version 0.8. 0.1. 2019.

[67] Canty A, Ripley B. Package ‘boot’ Bootstrap Functions version 1.3–27. 2021.

[68] Fox J, Weisberg S, Price B, Adler D, Bates D, Baud-Bovy G, et al. Package ‘car’ Companion to Applied Regression. 2020.

[69] Lenth VR, Buerkner P, Herve M, Love J, Riegel H, Singman H. R package “emmeans”: Estimated Marginal Means, aka Least-Squares Means. 2021.

[70] Barnier J, Briatte F, Larmarange J. questionr: Functions to Make Surveys Processing Easier. R package version 0.7. 0. 2018.

[71] Martin Maechler. Rmpfr: R MPFR—Multiple Precision Floating-Point Reliable 2021 [Available from: https://CRAN.R-project.org/package=Rmpfr].

[72] Katzmarzyk PT, Mason C. The physical activity transition. Journal of physical activity and health. 2009;6(3):269–80. doi: 10.1123/jpah.6.3.269 19564654

[73] Ng SW, Popkin BM. Time use and physical activity: a shift away from movement across the globe. Obesity reviews. 2012;13(8):659–80. doi: 10.1111/j.1467-789X.2011.00982.x 22694051 PMC3401184

[74] Guthold R, Stevens GA, Riley LM, Bull FC. Worldwide trends in insufficient physical activity from 2001 to 2016: a pooled analysis of 358 population-based surveys with 1· 9 million participants. The Lancet Global Health. 2018;6(10):e1077–e86. 30193830 10.1016/S2214-109X(18)30357-7

[75] Hallal PC, Andersen LB, Bull FC, Guthold R, Haskell W, Ekelund U, et al. Global physical activity levels: surveillance progress, pitfalls, and prospects. The lancet. 2012;380(9838):247–57. doi: 10.1016/S0140-6736(12)60646-1 22818937

[76] Sallis JF, Bull F, Guthold R, Heath GW, Inoue S, Kelly P, et al. Progress in physical activity over the Olympic quadrennium. The Lancet. 2016;388(10051):1325–36. doi: 10.1016/S0140-6736(16)30581-5 27475270

[77] Clark B, Sugiyama T. Prevalence, trends, and correlates of sedentary behavior. Physical activity, exercise, sedentary behavior and health: Springer; 2015. p. 79–90.

[78] Aekplakorn W. The fifth National Health Examination Survey (NHES V). Nonthaburi, Thailand: Health Systems Research Institute (HSRI); 2014.

[79] Aekplakorn W. The fourth National Health Examination Survey (NHES IV) 2008–2009. Nonthaburi, Thailand: National Health Examination Survey Office; 2009.

[80] Katewongsa P, Yousomboon C, Haemathulin N, Rasri N, Widyastari DA. Prevalence of sufficient MVPA among Thai adults: pooled panel data analysis from Thailand’s surveillance on physical activity 2012–2019. BMC public health. 2021;21(1):1–12. 33827516 10.1186/s12889-021-10736-6 PMC8028057

[81] Ramirez Varela A, Hallal P, Pratt M, Bauman A, Borges C, Lee I-M, et al. Global Observatory for Physical Activity (GoPA!): 2nd Physical Activity Almanac. Global Observatory for Physical Activity (GoPA!) [Internet]. 2021.

[82] Peltzer K, Pengpid S. Leisure time physical inactivity and sedentary behaviour and lifestyle correlates among students aged 13–15 in the association of southeast asian nations (ASEAN) member states, 2007–2013. International Journal of Environmental Research and Public Health. 2016;13(2). 10.3390/ijerph13020217 PMC4772237 26891312

[83] Institute for Population and Social Research. Final Report of ’Monitoring and Surveillance of Physical Activity in the Thai Population (2012–2014)’ (รายงานฉบับสมบูรณ์โครงการพัฒนาระบบเฝ้าระวังติดตามพฤติกรรมด้านกิจกรรมทางกายของประชากรไทย (2555–2557)). Nakhonpathom, Thailand: Institute for Population and Social Research, Mahidol University; 2016.

[84] National Statistical Office. The 2015 Physical Activity Survey. Bangkok, Thailand; 2016.

[85] Matthews CE, Keadle SK, Moore SC, Schoeller DS, Carroll RJ, Troiano RP, et al. Measurement of active and sedentary behavior in context of large epidemiologic studies. Medicine and science in sports and exercise. 2018;50(2):266–76. doi: 10.1249/MSS.0000000000001428 28930863 PMC5768470

[86] Ara I, Aparicio-Ugarriza R, Morales-Barco D, de Souza WN, Mata E, González-Gross M. Physical activity assessment in the general population; validated self-report methods. Nutricion hospitalaria. 2015;31(3):211–8. 25719788 10.3305/nh.2015.31.sup3.8768

[87] Gupta N, Mathiassen SE, Mateu-Figueras G, Heiden M, Hallman DM, Jørgensen MB, et al. A comparison of standard and compositional data analysis in studies addressing group differences in sedentary behavior and physical activity. International Journal of Behavioral Nutrition and Physical Activity. 2018;15(1):53. doi: 10.1186/s12966-018-0685-1 29903009 PMC6003121

[88] Lader D, Short S, J. G. The time use survey, 2005. London: Office for National Statistics; 2006.

[89] De Craemer M, De Decker E, De Bourdeaudhuij I, Vereecken C, Deforche B, Manios Y, et al. Correlates of energy balance‐related behaviours in preschool children: a systematic review. Obesity reviews. 2012;13:13–28. doi: 10.1111/j.1467-789X.2011.00941.x 22309062

[90] Temmel CS, Rhodes R. Correlates of sedentary behaviour in children and adolescents aged 7–18: A systematic review. The Health & Fitness Journal of Canada. 2013;6(1):119–99.

[91] Sigmundová D, Sigmund E. Weekday-Weekend Sedentary Behavior and Recreational Screen Time Patterns in Families with Preschoolers, Schoolchildren, and Adolescents: Cross-Sectional Three Cohort Study. International journal of environmental research and public health. 2021;18(9):4532. doi: 10.3390/ijerph18094532 33923313 PMC8123206

[92] Sigmundová D, Sigmund E, Badura P, Vokáčová J, Trhlíková L, Bucksch J. Weekday-weekend patterns of physical activity and screen time in parents and their pre-schoolers. BMC public health. 2016;16(1):1–9. doi: 10.1186/s12889-016-3586-8 27576897 PMC5004262

[93] Brooke HL, Corder K, Atkin AJ, van Sluijs EM. A systematic literature review with meta-analyses of within-and between-day differences in objectively measured physical activity in school-aged children. Sports Medicine. 2014;44(10):1427–38. doi: 10.1007/s40279-014-0215-5 24981243 PMC4171592

[94] Konharn K, Santos MP, Ribeiro JC. Differences between weekday and weekend levels of moderate-to-vigorous physical activity in Thai adolescents. Asia-Pacific Journal of Public Health. 2015;27(2):NP2157–NP66. doi: 10.1177/1010539512459946 23007484

[95] Drenowatz C, DeMello MM, Shook RP, Hand GA, Burgess S, Blair SN. The association between sedentary behaviors during weekdays and weekend with change in body composition in young adults. AIMS public health. 2016;3(2):375–88. doi: 10.3934/publichealth.2016.2.375 29546170 PMC5690362

[96] Kirk A, Gibson A-M, Laverty K, Muggeridge D, Kelly L, Hughes A. Patterns of sedentary behaviour in female office workers. AIMS public health. 2016;3(3):423–31. doi: 10.3934/publichealth.2016.3.423 29546173 PMC5689807

[97] Arifwidodo SD, Chandrasiri O. Association Between Park Characteristics and Park-Based Physical Activity Using Systematic Observation: Insights from Bangkok, Thailand. 2020;12(6):2559.

[98] Department of Health, Institute for Population and Social Research. Final Report of the Project on "Survey and Analysis of the Situation of Physical Activity Promotion in 5 Settings: Physical Activity Promotion Strategic Plans 2018–2030". Nakhonpathom, Thailand: Institute for Population and Social Research, Mahidol University; 2021.

[99] Michalchuk VF, Lee SJ, Waters CM, Hong OS, Fukuoka Y. Systematic Review of the Influence of Physical Work Environment on Office Workers’ Physical Activity Behavior. Workplace health & safety. 2022;70(2):97–119. doi: 10.1177/21650799211039439 35014589 PMC9733787

[100] Shrestha N, Kukkonen-Harjula KT, Verbeek JH, Ijaz S, Hermans V, Pedisic Z. Workplace interventions for reducing sitting at work. The Cochrane database of systematic reviews. 2018;6(6):Cd010912. doi: 10.1002/14651858.CD010912.pub4 29926475 PMC6513236

[101] Bellew B, Nau T, Smith BJ, Klepac Pogrmilovic B, Pedišić Ž, Bauman AE. Physical activity policy actions: What is the role of governments? In: Siefken K, Ramirez Varela A, Waqanivalu T, Schulenkorf N, editors. Physical activity in low- and middle-income countries. London, UK: Routledge; 2021. p. 44–62.

[102] Cooke JR, Ancoli-Israel S. Normal and abnormal sleep in the elderly. Handbook of clinical neurology. 2011;98:653–65. doi: 10.1016/B978-0-444-52006-7.00041-1 21056216 PMC3142094

[103] Ohayon MM, Carskadon MA, Guilleminault C, Vitiello MV. Meta-analysis of quantitative sleep parameters from childhood to old age in healthy individuals: developing normative sleep values across the human lifespan. Sleep. 2004;27(7):1255–73. doi: 10.1093/sleep/27.7.1255 15586779

[104] Biddle G, Edwardson C, Henson J, Davies M, Khunti K, Rowlands A, et al. Associations of Physical Behaviours and Behavioural Reallocations with Markers of Metabolic Health: A Compositional Data Analysis. International journal of environmental research and public health. 2018;15(10):2280. doi: 10.3390/ijerph15102280 30336601 PMC6210541

