# Supplementary material for: Physical activity, sedentary behaviour, and sleep in the Thai population: A compositional data analysis including 135,824 participants from two national time-use surveys
Source: PLoS One. 2023 Jan 24;18(1):e0280957. doi: 10.1371/journal.pone.0280957 (PMC9873167; doi:10.1371/journal.pone.0280957)
Supplement: S1 File — (PDF) [file pone.0280957.s001.pdf]

Variation matrix for the time-use composition

Time-use component	2009			2015		
	Physical activity	Sedentary behaviour	Sleep	Physical activity	Sedentary behaviour	Sleep
**Physical activity**	0	1.042	0.504	0	1.055	0.528
**Sedentary behaviour**	1.042	0	0.302	1.055	0	0.263
**Sleep**	0.504	0.302	0	0.528	0.263	0
